# Treatment of Ulcerative Colitis: Impact on Platelet Aggregation

**DOI:** 10.3390/medicina59091615

**Published:** 2023-09-07

**Authors:** Sasa Peric, Zeljko Todorovic, Nebojsa Zdravkovic, Andjela Gogic, Stefan Simovic, Vesna Grbovic, Mladen Maksic, Stefan Jakovljevic, Olivera Milovanovic, Natasa Zdravkovic

**Affiliations:** 1 Clinic of Gastroenterology and Hepatology, Military Medical Academy, Crnotravska Street 17, 11000 Belgrade, Serbia; pericsasa@gmail.com; 2 Department of Internal Medicine, Faculty of Medical Sciences, University of Kragujevac, Svetozar Markovic Street 69, 34000 Kragujevac, Serbia; simovicst@gmail.com (S.S.); asussonicmaster95@gmail.com (M.M.); natasasilvester@gmail.com (N.Z.); 3 Clinic for Hematology, University Clinical Center Kragujevac, Zmaj Jovina Street 30, 34000 Kragujevac, Serbia; 4 Department of Medical Statistics and Informatics, Faculty of Medical Sciences, University of Kragujevac, Svetozar Markovic Street 69, 34000 Kragujevac, Serbia; nzdravkovic@medf.kg.ac.rs (N.Z.); andjelica97@hotmail.com (A.G.); 5 Clinic for Cardiology, University Clinical Center Kragujevac, Zmaj Jovina Street 30, 34000 Kragujevac, Serbia; 6 Department of Physical Medicine and Rehabilitation, Faculty of Medical Sciences, University of Kragujevac, Svetozar Markovic Street 69, 34000 Kragujevac, Serbia; grbovicvesna72@gmail.com; 7 Center for Physical Medicine and Rehabilitation, University Clinical Center Kragujevac, Zmaj Jovina Street 30, 34000 Kragujevac, Serbia; 8 Clinic of Gastroenterology and Hepatology, University Clinical Center Kragujevac, Zmaj Jovina Street 30, 34000 Kragujevac, Serbia; 9 Department of Surgery, Faculty of Medical Sciences, University of Kragujevac, Svetozar Markovic Street 69, 34000 Kragujevac, Serbia; stefan_jakov87@yahoo.com; 10 Clinic for Surgery, University Clinical Center Kragujevac, Zmaj Jovina Street 30, 34000 Kragujevac, Serbia; 11 Department of Pharmacy, Faculty of Medical Sciences, University of Kragujevac, Svetozar Markovic Street 69, 34000 Kragujevac, Serbia; olivera.milovanovic09@gmail.com

**Keywords:** ulcerative colitis, platelet aggregation, infliximab, adalimumab, vedolizumab, azathioprine

## Abstract

*Background and Objectives*: Ulcerative colitis is chronic and/or progressive inflammation of the colorectal mucosa and submucosa and represents one of two major inflammatory bowel diseases. Ulcerative colitis has been associated with increased risk of arteriosus and venous thrombosis. There are numerous factors responsible for this; one of them is platelet activation and aggregation. The objective of our study was to determine if different treatment options for ulcerative colitis have an impact on platelet aggregation. *Materials and Methods*: This research was a prospective, observational study and included 94 newly diagnosed patients with UC divided into four treatment groups. For all patients, we measured platelet aggregability by using an impedance aggregometry method with a multiplate analyzer before and after treatment with infliximab, adalimumab, vedolizumab and azathioprine. A Paired Samples *t* test was performed in order to determine the difference in platelet aggregability before and after a certain therapy, since the data followed a normal distribution. Taking into account the impact of some clinical characteristics, multiple linear regression was conducted for the purpose of estimating the effect of therapy on the level of reduction in platelet aggregability. *Results*: All four drugs significantly reduced platelet aggregability. After we excluded the influence of clinical and endoscopic scores and disease localization on the results, we found that infliximab had the greatest anti-platelet activity. *Conclusions*: In addition to the well-known traditional risk factors for atherosclerosis, activation and aggregation of platelets play a significant role in the development of arterial thrombosis, and our results suggested that therapy use for the treatment of UC, especially infliximab, can have a great impact on cardiovascular morbidity and mortality by decreasing platelet aggregability.

## 1. Introduction

Ulcerative colitis (UC) is one of two major inflammatory bowel diseases (IBD). Ulcerative colitis is chronic and/or progressive inflammation limited to the colorectal mucosa and submucosa. Etiopathogenesis of UC has not been entirely defined, but it is known that major factors are genetic predisposition, environmental factors and inadequate immune response to these factors [1,2]. Worldwide, the incidence of UC is on the rise, ranging from 0.6 to 24.3 per 100,000 person-years in Europe and from 8.8 to 23.1 per 100,000 person-years in North America [3]. Incidence of UC is rising in many newly industrialized countries, which strongly implicates the role of environmental risk factors in the development of the disease [3].

For induction of UC remission, 5-aminosalicylic acid (5-ASA) drugs represent the standard therapy for patients with mild to moderate disease activity and corticosteroids for patients with moderate to severe UC or those who are unresponsive to standard therapy [4]. Thiopurines, such as azathioprine and mercaptopurine, were the first drugs used in maintenance of remission [4]. Today, there are many biological agents used in UC treatment. First, anti-tumor necrosis factor (TNF) drugs were introduced, including infliximab and adalimumab, and now, there are many agents like the anti-adhesion molecule vedolizumab, the interleukin inhibitor ustekinumab, the Janus kinase (JAK) inhibitor tofacitinib and many other therapeutic targets being explored in the treatment of UC in various clinical phases [1,5].

Ulcerative colitis has been associated with increased risk of arteriosus and venous thrombosis [6,7,8]. There are many factors contributing to thromboembolic events such as high levels of inflammatory cytokines [9] and, due to the systemic inflammatory response, increased factor VIII activity, elevated fibrinogen levels and accelerated thrombin generation [10]. Also, UC is associated with thrombocytosis due to the high plasma level of thrombopoietin and interleukin (IL)-6 [11]. Some studies found a correlation between thrombocytosis and atherosclerosis [12,13]. Aside from high platelet count, patients have also been found to have increased platelet activation and aggregation, which also increases the tendency for thrombosis in patients with UC [14,15]. There is evidence that treatment with adalimumab can also contribute to thromboembolic events [16].

The objective of our study was to determine if different treatment options for UC have an impact on platelet aggregation. As far as we know, this is the first study to show how different treatment options for UC impacts platelet aggregation.

## 2. Material and Methods

### 2.1. Patients and Treatment

A prospective, observational study was performed at the University Clinical Center Kragujevac, Center for Gastroenterohepatology and the Faculty of Medical Sciences, University of Kragujevac. All research procedures were sent to the Principle of Good Clinical Practice, and ethical approvals were obtained from relevant ethics committees.

A total of 94 newly diagnosed patients with UC were included in the trial. The patients were divided into four groups. All patients received induction treatment with only one drug, and platelet aggregability was measured before and 7 days after the induction treatment was finished. The first group had 27 patients, and they were treated with infliximab. Patients received infliximab intravenously with a dose of 5 mg/kg on the first day and the same dose after two weeks and after six weeks according to the induction protocol. The second group included 24 patients treated with adalimumab. Patients received 160 mg of adalimumab subcutaneously on the first day, 80 mg after two weeks and 40 mg after four weeks according to the induction protocol. The third group consisted of 19 patients who received vedolizumab intravenously, 300 mg on the first day and the same dose after two weeks and after six weeks. Lastly, the fourth group had 24 patients treated with azathioprine with a dose of 2–2.5 mg/kg administrated orally; after 14 weeks of treatment, we measured platelet aggregability in this group. Drug levels were measured after 7 days of the therapy and after the induction treatment was finished. All patients in this study had drug levels within the reference range, and no antibodies were detected. Patients with inadequate drug levels and the presence of antibodies were excluded from this study.

### 2.2. Inclusion and Exclusion Criteria

Participants had to meet the following inclusion and exclusion criteria.

#### 2.2.1. Inclusion Criteria

1. Diagnosis of UC made based on the endoscopic examination of the colon and the pathohistological findings of biopsies taken during the endoscopic examination in accordance with the criteria of the Third European Evidence-based Consensus on Diagnosis and Management of UC from 2017 [17];
2. Signed voluntary consent to participate in this study.

#### 2.2.2. Exclusion Criteria

1. Persons under 18 years of age, pregnant women, nursing mothers and persons with limited legal responsibility and reduced cognitive abilities;
2. Persons with chronic and malignant diseases and/or therapy that may affect the investigated parameters: antiplatelet, immunosuppressive, immunomodulatory and corticosteroid therapy;
3. Infections and infectious syndromes two months before and at the time of the research;
4. Patients positive to *Helicobacter pylori*, *Cytomegalovirus*, Hepatis B and C, and human immunodeficiency virus;
5. Patients with inadequate drug levels and presence of antibodies.

### 2.3. Platelet Aggregability

Heparinized whole blood samples collected before and 7 days after the last treatment were used to assess platelet aggregability. For platelet aggregability measurement, we used the impedance aggregometry method with a multiplate analyzer (Dynabyte, Munchen, Germany). This method is based on measuring the impedance between the two electrodes immersed into the whole blood sample after the addition of an aggregation agonist such as adenosine phosphate (ADP test), arachidonic acid (ASPI test) and thrombin receptor-activating protein (TRAP test) [18].

### 2.4. Patient’s Clinical end Endoscopic Characteristics

The clinical activity of the UC was determined according to the Truelove and Witts index [19], and the endoscopic disease activity was evaluated with the Mayo endoscopic subscore [20]. The Montreal classification of the extent of UC was used to classified patients into three subgroups [21].

### 2.5. Statistical Analysis

Statistical analysis was performed using IBM SPSS program version 22 (IBM SPSS Statistics 22. Armonk, NY, USA)**.** Distribution of categorical variables was shown as absolute (N) and relative (%) frequency. The normality of the distribution was determined using the Shapiro–Wilk test. Since the data followed a normal distribution, in order to determine the difference in the level of reduction in aggregation before and after a certain therapy, a Paired Samples t test was performed. For the purpose of estimating the effect of therapy on the level of reduction in platelet aggregability (ADP, ASPI and TRAP), taking into account the impact of some clinical characteristics, multiple linear regression was conducted.

## 3. Results

This study included 94 patients, divided into four groups based on the applied therapy. Their clinical and endoscopic characteristics are shown in Table 1.

**Figure 1:**
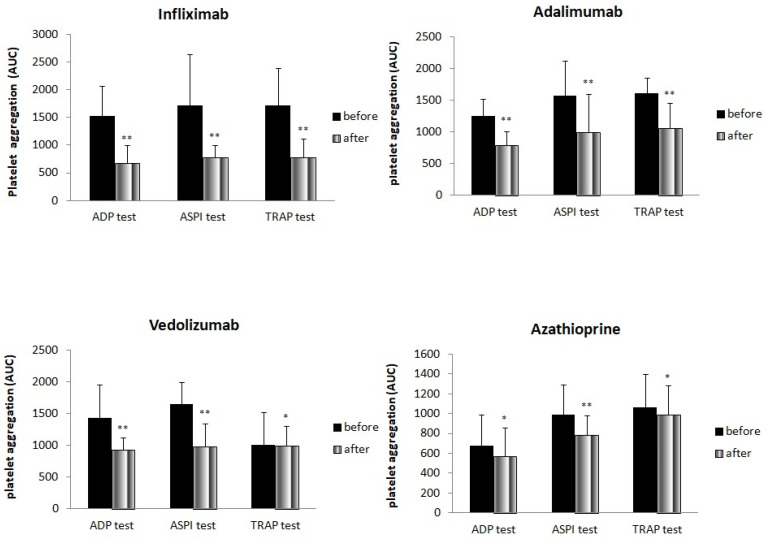
Platelet aggregability (ADP, ASPI and TRAP) before and after treatment (* *p* < 0.05, ** *p* < 0.01).

Using the impedance aggregometry method, we analyzed the impact of the four drugs on platelet aggregability. As it is shown in Figure 1, treatment with infliximab significantly reduced aggregability measured with all three tests, ADP, ASPI and TRAP. Similar results were found after treatment with adalimumab. Vedolizumab also showed a significant reduction in platelet aggregability using all three tests, with the smallest difference observed using the TRAP test. Azathioprine treatment also significantly reduced platelet aggregability measured with all three tests. However, the ASPI test showed the greatest reduction (Figure 1).

Multiple linear regression was performed to evaluate the effect of therapy on the reduction in platelet aggregability. Considering the heterogeneity of the groups in terms of clinical characteristics (clinical and endoscopic scores and disease localization), the model was adjusted for the aforementioned characteristics. The results showed that all four treatments significantly reduced platelet aggregation measured with the ADP and ASPI tests but not with the TRAP test. The greatest reduction in platelet aggregation was observed in the group with infliximab, while the lowest level of reduction was detected in the group treated with azathioprine (Table 2).

## 4. Discussion

Ulcerative colitis diseases can exhibit a wide range of extraintestinal manifestations, including cardiovascular ones [22]. The frequency of arterial and venous thromboembolism is 1.7–5.5 times higher than that in the general population [23]. Arterial thromboembolic events are most common in the active phase of UC. The most common manifestations are acute myocardial infarction, stroke and mesenteric ischemia [24].

In addition to the well-known traditional risk factors for atherosclerosis, activation and aggregation of platelets play a significant role in the development of arterial thrombosis. Under pathological conditions, platelets are able to adhere to the endothelium in the absence of endothelial denudation [25]. After initial contact, platelets contribute to the initiation and progression of atherosclerotic lesions via the release of granule proteins and microparticles, interaction with oxidized low-density lipoprotein (oxLDL) and crosstalk with immune and endothelial cell [26]. In animal models, aspirin and clopidogrel showed inhibitory effects on the progression of atherosclerotic lesions accompanied by a reduction in vascular inflammation, decreased serum levels of inflammatory markers, reduced NFκB activity and a more stable plaque phenotype [27,28]. These data suggested that anti-platelet drugs have anti-inflammatory properties and inhibit atherosclerosis.

In our research, we used anti-TNF drugs infliximab and adalimumab; the anti-adhesion drug vedolizumab; and one immunosuppressive drug, azathioprine. By using the impedance aggregometry method with a multiplate analyzer (ADP, ASPI and TRAP tests) we measured the impact of these drugs on platelet aggregation. In the ADP test, platelets were activated by ADP, which triggers several receptors on the platelet surface. clopidogrel and related drugs blocked the P2Y12 receptor, which is believed to be the most important receptor for ADP on the platelet surface. In the ASPI test, platelets are activated by arachidonic acid. Further on, platelet cyclooxygenase enzyme (COX-1) converts the arachidonic acid to the potent platelet agonist thromboxane A2. Arachidonic acid alone is not a platelet agonist and is the physiological substrate of the platelet COX-1. Therefore, platelet activation in the ASPI test allows very sensitive and specific detection of acetylsalicylic acid action. In the TRAP test, platelets are activated by TRAP-6, a peptide that mimics the activation of platelets via the action of thrombin. Thrombin is the most potent platelet activator. The action of thrombin is not blocked by the antiplatelet agents—acetylsalicylic acid or clopidogrel. However, thrombin (and TRAP-6)-activated platelets are blocked by the action of GPIIb/IIIa antagonists [18]. These tests are widely used to evaluate non-responders to acetylsalicylic acid and clopidogrel therapy with high accuracy [29,30]. Our investigated drugs are not blockers of P2Y12 receptor, nor of COX-1 and GPIIb/IIIa, so all three tests were equal in assessing platelet aggregation.

All four drugs showed significant anti-platelet activity, and platelet aggregation measured with the ADP, ASPI and TRAP tests was reduced significantly. Compared with each other, infliximab was the drug with the greatest anti-platelet activity and azatioprin showed the least anti-platelet activity. Because our treatment groups were heterogeneous in terms of clinical and endoscopic scores and disease localization and all these factors could influence platelet aggregability, we excluded the influence of these factors on results using regression analyses. We found that all four treatments significantly reduced platelet aggregation measured with the ADP and ASPI tests but not with the TRAP test, but infliximab still showed the greatest reduction in platelet aggregability and azatioprin showed the least reduction.

These results correlate with a previous study that showed that platelet activity was reduced in patients with rheumatoid arthritis treated with TNFα inhibitors. Also, previous studies have shown that TNFα antagonists are associated with risk reduction in cardiovascular events among patients with rheumatoid arthritis, psoriasis and psoriatic arthritis [31,32,33]. Tumor necrosis factor α contributes to the risk of cardiovascular disease by promoting platelet hyperreactivity due to reprogramming of the inflammatory, mitochondrial and metabolic pathways in megakaryocytes [34]. Perhaps the better results obtained with the use of infliximab compared to adalimumab are due to its intravenous administration, but this remains a topic for additional research.

Histopathological studies found mesenteric vascular microthrombi to be the first finding in the mucosa of UC patients [35]. Those microthrombi contribute to ischemia so our study suggested that all four drugs could reduce severity of UC by reducing thrombosis events in colon mucosa.

Our study was unique because, for the first time, it showed how different treatments for UC influence platelet aggregability. These results suggested that therapy for the treatment of UC, especially infliximab, not only reduces the activity of the disease but also can have a great impact on cardiovascular morbidity and mortality.

## 5. Conclusions

In addition to the well-known traditional risk factors for atherosclerosis, activation and aggregation of platelets play a significant role in the development of arterial thrombosis and our results suggested that therapy for treatment of UC can decrease platelet aggregability.

## Figures and Tables

**Table 1:** Patient’s clinical and endoscopic characteristics.

Patient Characteristics		Infliximab		Adalimumab		Vedolizumab		Azatioprine	
		*N*	%	*N*	%	*N*	%	*N*	%
clinical score	mild	0	0.0	1	4.2	0	0.0	10	41.7
	moderate	13	48.1	11	45.8	15	78.9	10	41.7
	severe	14	51.9	12	50.0	4	21.1	4	16.7
endoscopic score	normal	0	0.0	0	0.0	0	0.0	0	0.0
	mild	0	0.0	0	0.0	0	0.0	9	37.5
	moderate	15	55.6	13	54.2	16	84.2	12	50.0
	severe	12	44.4	11	45.8	3	15.8	3	12.5
disease localization	proctitis	0	0.0	0	0.0	1	5.3	0	0.0
	left-sided colitis	8	29.6	12	50.0	12	63.2	18	75.0
	pancolitis	19	70.4	12	50.0	6	31.6	6	25.0

**Table 2:** Effect of therapy on the level of reduction in platelet aggregability (ADP, ASPI and TRAP), adjusted for clinical and endoscopic scores and disease localization (* *p* < 0.05, ** *p* < 0.01).

Treatment	Difference in ADP before and after Treatment		Difference in ASPI before and after Treatment		Difference in TRAP before and after Treatment	
	B	95% CI	B	95% CI	B	95% CI
Infliximab	854.289 **	656.56–1052.02	517.303 **	276.45–758.16	166.934	−82.88–416.75
Adalimumab	351.064 *	146.30–555.83	308.267 *	58.84–557.69	71.881	−186.83–330.59
Vedolizumab	311.067 *	118.92–503.21	327.070 *	93.01–561.13	136.894	−379.66–105.87
Azatioprine	178.365 *	85.25–268.75	212.214 *	32.51–452.37	59.856	−153.98–254.40

## Data Availability

The data presented in this study are available from the corresponding author upon request.
